# Targeting of *De Novo* DNA Methylation Throughout the *Oct-4* Gene Regulatory Region in Differentiating Embryonic Stem Cells

**DOI:** 10.1371/journal.pone.0009937

**Published:** 2010-04-01

**Authors:** Rodoniki Athanasiadou, Dina de Sousa, Kevin Myant, Cara Merusi, Irina Stancheva, Adrian Bird

**Affiliations:** Wellcome Trust Centre for Cell Biology, University of Edinburgh, Edinburgh, United Kingdom; CNRS, France

## Abstract

Differentiation of embryonic stem (ES) cells is accompanied by silencing of the *Oct-4* gene and de novo DNA methylation of its regulatory region. Previous studies have focused on the requirements for promoter region methylation. We therefore undertook to analyse the progression of DNA methylation of the ∼2000 base pair regulatory region of *Oct-4* in ES cells that are wildtype or deficient for key proteins. We find that de novo methylation is initially seeded at two discrete sites, the proximal enhancer and distal promoter, spreading later to neighboring regions, including the remainder of the promoter. De novo methyltransferases Dnmt3a and Dnmt3b cooperate in the initial targeted stage of de novo methylation. Efficient completion of the pattern requires Dnmt3a and Dnmt1, but not Dnmt3b. Methylation of the *Oct-4* promoter depends on the histone H3 lysine 9 methyltransferase G9a, as shown previously, but CpG methylation throughout most of the regulatory region accumulates even in the absence of G9a. Analysis of the *Oct-4* regulatory domain as a whole has allowed us to detect targeted de novo methylation and to refine our understanding the roles of key protein components in this process.

## Introduction

Approximately 90% of all CpGs in the mammalian genome are methylated at the 5 position of the cytosine ring. Specific cell types and tissues have signature DNA methylation patterns [1]–[4] that arise during development in the differentiating cell types [5], [6]. Despite the consistency of the methylation patterns in different cell types and an apparent developmental program for the transition from one methylation state to another, little is known about the detailed biological mechanisms by which DNA methylation patterns are established. Several key proteins that affect this epigenetic modification are known; most importantly the DNA methyltransferases, Dnmt1, Dnmt3a and Dnmt3b. Dnmt1 is the “maintenance methyltransferase” that localizes to replication foci during S phase [7] and copies the DNA methylation pattern to the newly synthesized daughter strand. Further support to this view comes from *in vitro* demonstrations that Dnmt1 preferentially methylates hemimethylated DNA [8]. Dnmt3a and Dnmt3b, on the other hand, are *de novo* methyltransferases, responsible for the methylation of unmodified DNA. Disruption of all three *Dnmt* genes in mouse embryonic stem (ES) cells abolishes CpG methylation [9] demonstrating that CpG methylation is exclusively dependent on these enzymes. Interestingly, knock-outs of other protein coding genes, including *G9a* and *Lsh*, also reduce global DNA methylation levels [10], [11].

Little is known about the relative contribution of Dnmt3a and Dnmt3b to *de novo* methylation patterns. Deletion of the catalytic activities of either enzyme showed that, at the majority of the studied loci, methylation was not affected [12]. Only when both enzymes were depleted could the DNA methylation be erased at these loci. This suggests that, in most cases, the two enzymes complement one other. This is further supported by the fact that Dnmt3a and b associate with one another [13]. There are, however, differences in specificity, as Dnmt3b alone has been shown to be responsible for the methylation of centromeric minor satellite repeats [12], whereas Dnmt3a alone is able to restore the methylation in the *Xist* and *H19* loci in cells carrying inactivating mutations in both enzymes [14]. *In vitro* experiments have not revealed intrinsic sequence specificities of the Dnmt3 enzymes and more in vivo studies are needed to dissect the roles of the two proteins in *de novo* methylation of individual genes.

It seems likely that local DNA methylation patterns arise not from an intrinsic specificity of Dnmts themselves, but via interactions with other DNA binding proteins. Transcription factors in particular are known to display DNA sequence specificity and Dnmts have been reported to associate with E2F-Rb [15], GCNF [16], COUP-TF1[17], PML-RAR [18] and RP58 [19]. Dependence of DNA methylation on histone modifications has been clearly demonstrated in fungi and plants [20]–[23], but in animals this link is less robust. Nevertheless, there is evidence that the histone H3 lysine 9 methylatransferase G9a can recruit Dnmts to the *Oct-4* locus and other loci upon ES cell differentiation [10].

Local exclusion of DNA methylation represents another general mechanism for determining patterns of DNA methylation and this can also depend on transcription factor binding. The non-methylated status of the CpG island at the rodent *aprt* gene, for example, depends on the presence of Sp1 binding sites in the promoter of the gene [24]–[26], although the mechanism of protection is unknown. Evidence for similar prevention of DNA methylation has also been uncovered at the imprinted *H19/Igfr2r* locus [27]. In that study, binding of the CTCF factor to the differentially methylated region (DMR) of the maternal allele appeared to prevent methylation and regulate enhancer activity in *cis*.

In this study we revisit the *in vitro* differentiation of embryonic stem (ES) cells in order to study the establishment of DNA methylation in the upstream regulatory region of the *Oct-4* gene. Previous high-resolution studies have focused on the *de novo* methylation of the promoter region of *Oct-4* [28]–[32], but have not analyzed parameters that influence methylation of the 2000 base pair upstream region that has been implicated in the differential regulation of Oct-4 gene expression in ES cells and the epiblast [33]. We therefore decided to establish the detailed dynamics of methylation at all known regulatory elements of the gene using both wildtype and mutant ES cell lines. Our findings uncover targeted *de novo* methylation followed by spreading throughout the region. In addition, we detect differential roles for Dnmt3a and Dnmt3b in the spreading phase and implicate Dnmt1 and Lsh in this process. Finally, we find that the histone H3K9 methyltransferase G9a is dispensable for methylation of much of the Oct4 regulatory region.

## Results

### DNA methylation is initiated at discrete regions of the *Oct-4* regulatory region

**Figure 1:**
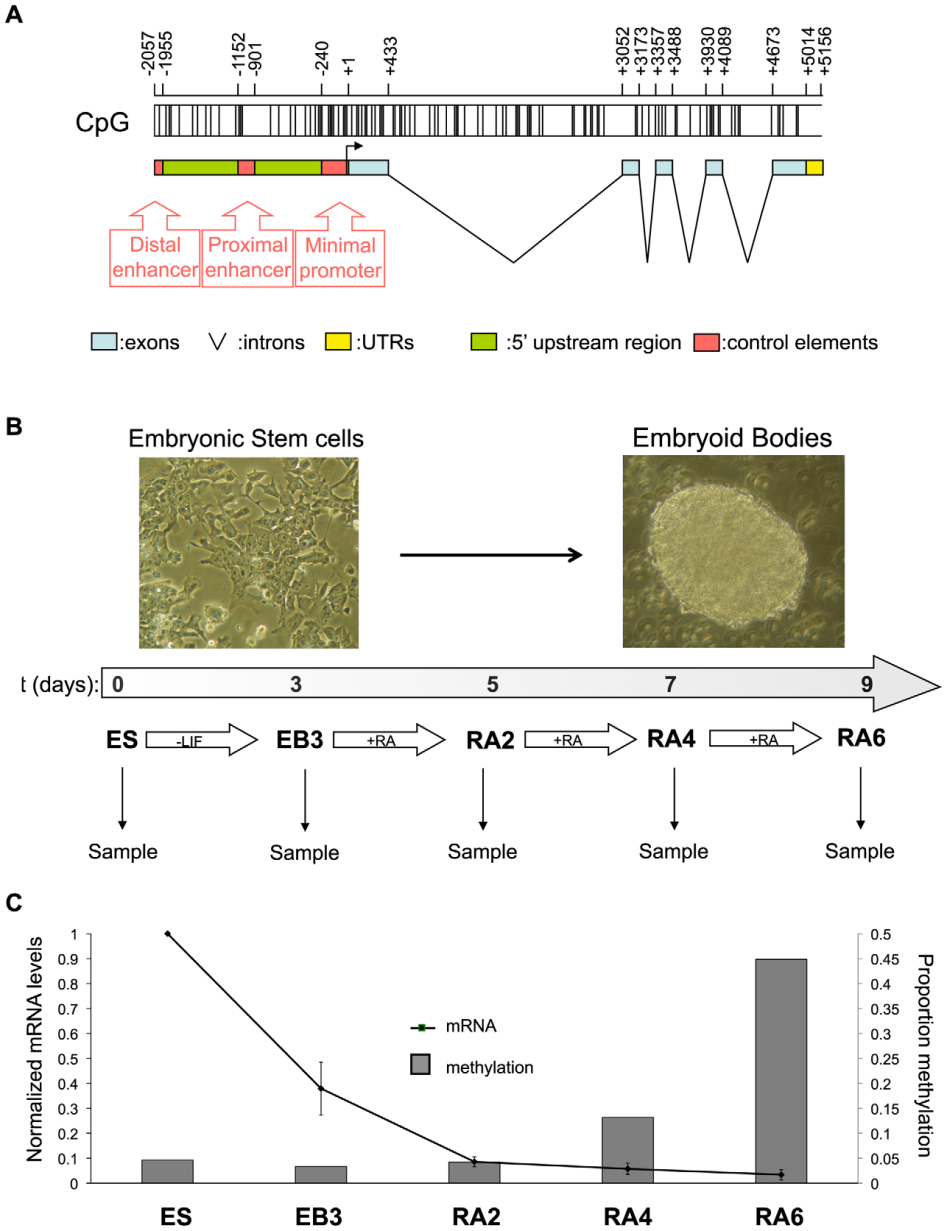
Acquisition of DNA methylation within the upstream regulatory region of the mouse *Oct-4* gene. **A**) Schematic diagram of the *Oct-4* upstream regulatory region. **B**) Outline of the protocol for ES cell *in vitro* differentiation. **C**) Expression and methylation profiling of *Oct-4* during *in vitro* differentiation of wildtype ES cells. The expression is the average of four independent experiments and the error bars show ± the standard error of the mean. Methylation values are the average for the entire upstream region (see detailed bisulfite results in Figure 2).

**Figure 2:**
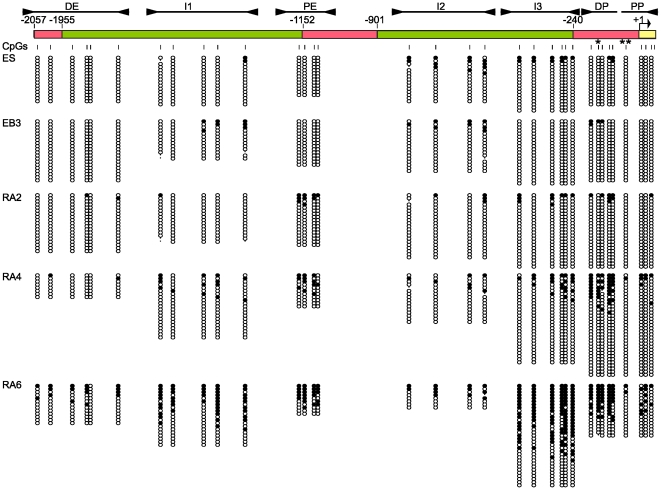
Time-course of DNA methylation across the upstream regulatory region of *Oct-4* during *in vitro* differentiation. Convergent arrowheads indicate the position of the primer pairs and solid lines the segments analysed. Each differentiation stage is indicated at the left with the corresponding methylation data to its right. Empty and filled circles denote unmethylated and methylated CpGs, respectively. Each horizontal row of circles represents one sequenced DNA clone. The single asterisked CpG indicates the HpyCh4 IV recognition site and the double asterisked CpG indicates the RARE that is always protected (see text).

Three regulatory elements, corresponding to the promoter, the proximal enhancer and the distal enhancer, have been identified in the 2Kb region upstream of the transcription start site of *Oct-4* (Figure 1A) [33]–[35]. In order to dissect the establishment of methylation in the upstream region of the gene, mouse ES cells were differentiated *in vitro* for nine days: LIF was removed from the medium on day 1 and, 3 days later, retinoic acid (RA) was added for up to 6 days. Cells were harvested at the following stages: undifferentiated ES cells (ES), embryoid bodies after 3 days of differentiation (EB3), differentiating cells after 2, 4 or 6 days of treatment with retinoic acid (RA2, RA4 and RA6) for mRNA and DNA methylation analysis (Figure 1B). In accordance with previous studies of a subset of CpGs in the 2 Kb upstream region of the gene [30], the methylation levels of the entire 2 Kb upstream regulatory region of *Oct-4* in our experimental system increased only after transcription of the gene had been silenced (Figure 1C). Inspection of the detailed methylation profile of the region confirmed that no CpG position had accumulated appreciable methylation levels before the RA2 stage (Figure 2), by which point the gene was virtually silent. These findings confirm that DNA methylation is not responsible for the primary silencing event at this gene.

We wanted to know if the DNA methyltransferases responsible for the observed methylation of the *Oct-4* locus upon *in vitro* differentiation were preferentially targeted to specific sites within this domain or if the process stochastically affected all CpGs equally. In the first case we should be able to observe distinct methylation foci, while in the second, methylation should uniformly increase throughout the examined region. We divided the 2 kb upstream region in seven segments (Figure 2, top), four of which overlapped the known regulatory elements of *Oct-4*: the distal enhancer (DE); the proximal enhancer (PE), and the distal and proximal regions of the promoter (DP and PP). In addition, we analysed three intergenic regions with no known regulatory function (I1, I2 and I3). Although the entire promoter region has promoter activity in reporter assays [33], only the proximal portion contains known transcription factor response elements, anchors of the basal transcription machinery and is conserved in mammals [34]. Altogether our analysis includes all 34 CpG sites within 2057 bp upstream of the transcription start site.

**Figure 3:**
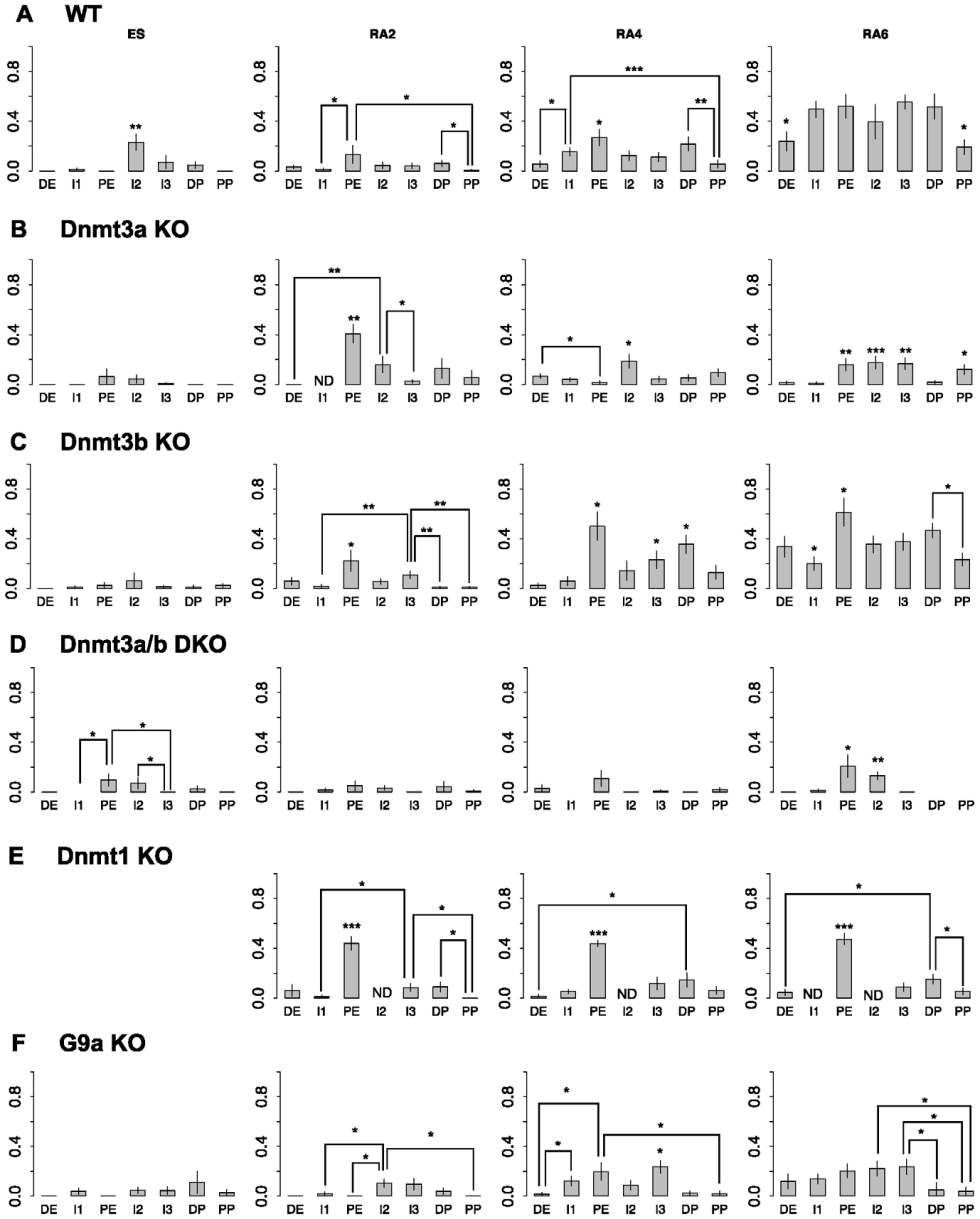
Altered DNA methylation in differentiating ES cells lacking proteins implicated in *de novo* methylation. The y-axis shows the ratio of methylated CpGs versus all CpGs in each segment. The differentiation stages are shown above each column and genotypes are indicated on the left. ND, not determined. The error bars show the standard error of the mean and asterisks denote the p-value calculated using the Exact Wilcoxon test. Only significant values according to the Wilcoxon permutation test are shown using the following convention: *, p<0.05; **, p<0.01; ***, p<0.001. Similar trends in pairwise statistical significance were summarized by asterisks above a single bar. In these cases only the lowest p value of all pairwise comparisons is shown. A detailed list of the pairwise comparisons that have been summarized in this figure is found in the Text S1.

Two days after addition of RA (RA2), DNA methylation became detectable at the proximal enhancer and distal promoter regions (Figure 3). Methylation in these regions continued to increase at RA4, while the distal enhancer and proximal promoter regions appeared comparatively resistant to methylation by comparison. Preferential appearance of methylation peaks at PE and DP was confirmed in independent experiments (Figure S1A-B), while the absence of any corresponding peak in a random in silico-generated methylation pattern, matching the experimental data for the number of CpGs and the number of clones sequenced per segment, showed that the observed pattern is unlikely to be an artifact of the experimental design (Figure S1C). By the end of the differentiation process (RA6), methylation was high throughout the region, although the DE and PP continued to have significantly lower methylation levels. A similar pattern to RA6 was revealed when DNA from adult mouse tail tips were analyzed (Figure S1D), indicating that RA6 successfully captured the end-point of the methylation process. As an independent method of assessing DNA methylation we performed COBRA analyses at the distal promoter region of *Oct-4*. This agreed with the methylation levels as measured by bisulfite sequencing (Figure S2).

### Dnmt3a, Dnmt3b and Dnmt1 have overlapping but distinct roles in the establishment of the methylation pattern

Our data show that DNA methylation is initially targeted to the proximal enhancer and distal promoter elements of the *Oct-4* promoter. To assess the specific contribution of each Dnmt in the establishment of the methylation pattern, we repeated the *in vitro* differentiation process (Figure 1B) with ES cells that were null for *Dnmt3a*, *Dnmt3b*, both *Dnmt3a* and *Dnmt3b*, or *Dnmt1*. Although these cells have been reported to be defective in the differentiation process [36],we found that the silencing of the Oct-4 gene under the influence of retinoic acid in both *Dnmt1* and *Dnmt3a/b* mutants followed kinetics that were indistinguishable from wildtype ES cells (Figure S3 B-C). We therefore consider that the differentiation defects of these mutants do not affect to the events that accompany *Oct-4* silencing.

In the absence of Dnmt3a (Figure 3B), which was previously shown to be enriched in the promoter of *Oct-4* upon differentiation [31], the initial phase of *de novo* methylation appeared relatively normal, a pronounced peak appearing at PE. As differentiation progressed, however, the cells were unable to maintain the methylation pattern and by the end of the differentiation process the overall methylation level was low and distributed across the region. Especially for the PE we observed a drop of the methylation levels between RA2 and RA4 that cannot be explained by passive demethylation of the locus. A possible explanation is that there has been some random clonal selection on the population of differentiating ES cells. These results indicate that Dnmt3b can initiate DNA methylation at this locus but cannot alone complete the process. Absence of Dnmt3b, on the other hand, had no significant effect on the methylation pattern and preserved the preferential methylation of PE and DP, as in the wildtype cells (Figure 3C). We conclude that although *Dnmt3b* may contribute to the initiation of methylation at the locus, it is not essential for this process.

The *Dnmt3a/3b* double knock-out cell line (Figure 3D) had a more severe methylation phenotype than the *Dnmt3a* KO, as initiation of methylation at PE and DP was not seen and low methylation levels did not change markedly during differentiation. The initiation and spreading of DNA methylation at the *Oct-4* regulatory region is therefore dependent on *de novo* methyltransferases and cannot be achieved by the “maintenance” enzyme Dnmt1 alone. Deletion of *Dnmt1* is incompatible with differentiation and reduces the global DNA methylation levels to about 20% of wildtype [36]. Nevertheless, a prominent peak of methylation was present at PE in these cells (Figure 3E). Thus in the presence of Dnmt3a and 3b alone, the proximal enhancer remains a target for de novo methylation. For this to spread to other parts of the regulatory region, however, Dnmt1 is required.

### G9a is important for the establishment of the methylation pattern but not for the recruitment of Dnmts

It has been reported that DNA methylation at the Oct-4 promoter is dependent on recruitment of the histone H3 lysine 9 methyltransferase G9a. We therefore asked whether the same G9a dependence applied to the entire regulatory region of *Oct-4* (Figure 3F). Quantitative expression analysis confirmed that *Oct-4* expression declines during differentiation, as in wildtype ES cells (Figure S3D). We confirmed the previous finding that promoter methylation is minimal in *G9a*-null ES cells [31], as methylation at RA6 was present at only about 5% of CpGs in PP and DP. Unexpectedly, extensive de novo methylation accrued elsewhere in the regulatory region as these mutant cells differentiated. The overall level of methylation across the examined domain reached ∼20% of CpGs by RA6 compared with ∼40% in wildtype cells. These results show that G9a is important in the formation of the wildtype Oct-4 methylation pattern, but significant levels of methylation do accumulate in its complete absence.

### Lsh improves coordinated methylation of neighbouring CpGs

**Figure 4:**
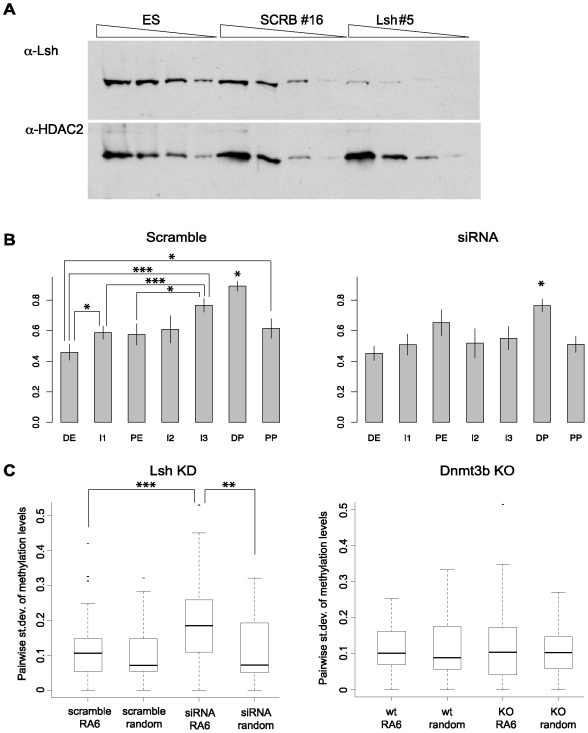
Nearest neighbour DNA methylation analysis reveals a defect in Lsh-deficient ES cells. (**A**) Western blot of the parental wt ES cell line, as well as the scramble and Lsh KD cell lines used in this study. TOP: anti-LSH antibody, BOTTOM: loading control, anti-HDAC2 antibody (**B**) DNA methylation levels in each segment of the *Oct-4* regulatory region in cells harboring the specific construct against Lsh (siRNA) or a scrambled control (scramble). The RA6 time point was analyzed. Annotation is as Figure 3. The asterisks above DP in both bar plots are against all other segments. (**C**) Box plots of the standard deviations of the methylation levels of neighboring CpGs. The statistical analysis was performed with the Wilcoxon test.

Mammalian Lsh is closely related to the Arabidopsis protein DDM1 and mutations in both genes cause hypomethylation of the genome [37], [38]. Lsh belongs to the SNF2 family of chromatin-remodeling ATPases and has been shown to interact with Dnmt1, Dnmt3a and Dnmt3b [11], [39]. Lsh has been reported to play a role in the establishment of methylation in at least one CpG in the promoter of *Oct-4* (HpyCH4 IV site, see asterisk in Figure 2) [11]. We therefore sought to analyze in detail the effect of Lsh deficiency on the establishment of DNA methylation throughout the upstream regulatory region of *Oct-4*. For this, we created stable ES cell lines containing the siRNA construct for Lsh used in previous studies [11] and after identification of the successful knock-downs (Figure 4A), we differentiated them as described in Figure 1B. In agreement with previous results [11], at RA6 we observed a moderate reduction in the methylation levels at the HpyCh4 IV site (52% in the siRNA relative to 83% in scramble). Analysis of the bisulfite results segment-by-segment, however, showed that the effect of the Lsh depletion on DNA methylation levels and the overall methylation pattern was minimal (Figure 4B). Closer examination did however reveal a difference in the coherence of DNA methylation at adjacent CpG sites (Figure S4). As a measure of variability, we calculated the standard deviations of the methylation levels of each pair of neighboring CpGs in the *Oct-4* upstream region for the cells treated with siRNA and a scrambled sequence control. An additional control was generated *in silico* as a randomized methylation pattern matched for the number of clones sequenced and number of sequencing amplicons in the experiment. As Figure 4B shows, the RA6 sample with downregulated Lsh had significantly less coordination of DNA methylation between adjacent sites than did the scrambled siRNA samples or the randomly generated control pattern. Moreover, this result was specific to Lsh knockdown, as it was not reproduced in RA6 samples from *Dnmt3b*-null cells (Figure 4C, right panel). A possible interpretation of our data is t that the motor function of Lsh contributes to the processivity of de novo methylation by insuring that modification of a CpG site leads to modification also at adjacent CpGs in the genomic DNA sequence.

## Discussion

Despite the undisputed importance of DNA methylation establishment in mammalian development, little is known about the mechanism that targets specific loci in the genome in a timely and coordinated manner. A key reason for our ignorance has been the scarcity of known genomic loci that reproducibly acquire DNA methylation within a defined timeframe. An exception is the *Oct-4* gene, which has been the subject of several studies. We decided to re-visit this system in order to extend the analysis to all CpG sites within the 2000 bp regulatory region that drives regulated expression of this gene. This region contains the three regulatory elements (the promoter, the proximal enhancer and the distal enhancer) that have been shown to regulate *Oct-4* expression *in vivo* [35].Our findings confirmed that DNA methylation of the region follows silencing of the Oct-4 gene. We also verified that the histone methyltransferase G9a is required for appropriate methylation of the promoter region, but the data show that methylation of most of the region is not G9a-dependent. The proposed central role of G9a in transcriptional shut down at this locus deserves reassessment in the light of these data.

We detected a distinctive methylation pattern during the early stages of establishment of DNA methylation at the upstream regulatory region of the *Oct-4* gene. The proximal enhancer and, to a lesser extent, the distal portion of the promoter are the primary targets for methylation, which subsequently spreads and accumulates throughout the region. The distal enhancer and the proximal promoter, however, maintain significantly lower methylation levels throughout, suggesting that they represent the boundaries of the DNA methylation domain. An important implication of this observation is that the Dnmts responsible for methylating this region do not attack the silenced gene at random but are targeted to specific locations. A previous study of de novo methylation at the human *P16* gene in primary mammary epithelial cell lines inferred site-specific initiation of de novo methylation followed by spreading to intervening regions [40].

The mechanism of targeting to DP and PE is not known, but a simple possibility is that the enzymes are actively recruited to these sites once silencing has occurred. Supporting this model, the transcriptional activator LRH-1 is released from the early-methylated PE upon differentiation as part of the gene-silencing process [41]. We also note that the retinoic acid response element (RARE) in the promoter (double-asterisk Figure 2) is the anchoring site for both the *Oct-4* activator SF-1 and the repressor GCNF [42]. This CpG site is therefore expected to be protected whether the gene is active or inactive, which may explain why it is always hypo-methylated relative to surrounding CpGs in this (Figure 3A) and other [30] studies. The repressor GCNF has been implicated in active recruitment of Dnmts. Co-immunoprecipitation assays showed interaction with Dnmt3a and Dnmt3b [30] and overexpression of either GCNF alone, or GCNF plus Dnmt3a, increased the methylation levels of DP. In the absence of GCNF there is virtually no methylation at the I3, DP and PP regions upon differentiation of ES cells [28]. Taken together, these results suggest a mechanism whereby specific recruitment of Dnmts and accessibility to specific DNA sequences combine to generate the methylation pattern identified in this study.

We analyzed the specific contribution of each Dnmt to the methylation pattern by using mutant ES cell lines. Although these experiments demonstrated redundancy in the activity of Dnmt3a and Dnmt3b as regards initiation of de novo methylation, on the *Oct-4* upstream regulatory region, they indicated that Dnmt3a plays a dominant role over Dnmt3b during the spreading phase. In the absence of Dnmt3b, the level of methylation in the Oct-4 regulatory region was minimal after 6 days treatment with retinoic acid. A previous study also showed a more severe effect in the methylation of the promoter and I3 regions of the *Oct-4* gene in the absence of Dnmt3a than Dnmt3b [29]. In the same study however, absence of DNMT3b had an appreciable effect on the methylation levels, which nevertheless varied depending on the examined cell type. Examination of the 2 kb upstream regulatory region in the Dnmt3a/b double knockout cell line also suggested a role of Dnmt1 in spreading, as the accumulation of DNA methylation outside PE and DP was negligible during days 2 to 6 of retinoic acid treatment in Dnmt1-deficient cells. Others have observed that the *Oct-4* promoter in *Dnmt1*-/- fibroblasts is less methylated than in wild-type [31]. Although Dnmt1 is regarded as having exclusively maintenance methyltransferase activity, previous studies have shown it can cooperate with Dnmt3a for *de novo* methylation of both naked [43] and nucleosomal [44] DNA. Furthermore, Dnmt3 proteins co-immunoprecipitate with Dnmt1 [13], [45]. Our results support an active role of Dnmt1 for *de novo* methylation pattern establishment.

Studies of a subset of CpGs that coincide with methylation-sensitive restriction sites in the promoter of *Oct-4* have suggested a targeting role for the H3K9 methyltransferase G9a in recruiting Dnmts to *Oct-4* [31]. Our bisulfite results support the conclusion that G9a is essential for full methylation of the promoter, in agreement with previously published bisulfite data on the promoter of the gene [10], but it is clear that de novo methylation elsewhere within the 2 kb regulatory region can be established in the absence of G9a. Accumulation of methylation to wildtype levels is, however, incomplete in differentiating *G9a*-null cells, suggesting that the presence of G9a facilitates full methylation in this region.

The effect of Lsh depletion on overall DNA methylation levels in the Oct-4 regulatory region was negligible, but we found that the correlation between levels of methylation at adjacent CpG sites was significantly reduced. It should be noted that there were residual levels of Lsh in the stable KD cell lines, which could explain the subtlety of the observed phenotype. A potential explanation for this effect is that Lsh contributes to the cooperativity of DNA methylation spreading. When Lsh is depleted, CpG site methylation appears to be more stochastic as sites are methylated independently of the methylation status of their neighbors. As Lsh is related to ATP-dependent motor proteins, it is conceivable that it facilitates mobilization of Dnmts within this region of the genome. Consistent with this possibility, Lsh has been shown to interact with Dnmt1, Dnmt3a and Dnmt3b [11], [39]. The reduced genome-wide methylation observed in Lsh KO cells and tissues [38] may be explained by failure of Dnmt mobilization. Similarly, the residual methylation observed in the I2-I3 regions upon differentiation of Lsh knock-down EC cells [46], could also be explained by this mechanism. It has been shown that Lsh -/- Day 18.5 embryos have an 8-fold reduction in the methylation of the I1, PE and promoter regions of the *Oct-4* gene, which could be the result of long-term lack of Dnmt mobilization or Dnmt stability on the locus [46].

Overall, our results suggest that the Oct-4 regulatory region is best viewed as an extended DNA methylation domain whose de novo DNA methylation during differentiation occurs in two phases: initiation of methylation preferentially at specific sites, followed by spreading of methylation throughout the domain. Progression through these phases requires the collaboration of several proteins in addition to the *de novo* and maintenance Dnmts. Analysis of the kinetics and factor-dependency of epigenetic changes across the entire domain may permit a better understanding of the mechanisms underlying *de novo* methylation in general.

## Materials And Methods

### Cell lines and cell culture

The wt ES cell line used in the study was the E14Tg2α [47]. The Dnmt3a, Dnmt3b Dnmt3a/b knockout cell lines [12] and the Dnmt1^S^ KO cell line [48] are were gifts from Dr En Li. The G9a KO cell line is the 2–3 clone described in [49].

For the creation of stable knocked-down Lsh ES cell lines, the E14Tg2α ES cells were transfected with the siRNA plasmid [11] using Lipofectamine 2000 (Invitrogen). The cells were then plated at low densities and the successful transfections were selected with G418 for ten days. Single-cell colonies were picked and expanded under continuing G418 selection.

ES cells were grown on precoated gelatinized flasks at 37°C in the presence of 5% CO_2_ in 1x Glasgow modified Eagle's Medium (Invitrogen) supplemented with 1 mM sodium pyruvate (Invitrogen), 1x non-essential amino acids (Invitrogen), 10% foetal bovine serum (HyClone), LIF and 1∶1000 β-mercaptoethanol (Invitrogen).

For *in vitro* differentiation, one T75 flask of confluent ES cells was transferred to a 100 mm^2^ bacteriological Petri dish with full medium without LIF and incubated at 37°C in 5% CO_2_ for three days. Then a 1∶10,000 dilution of RA stock solution (Sigma) was added to the full medium without LIF and the cells were harvested after 2, 4 or 6 days. The differentiation medium was changed every two days and the developing embryoid bodies were handled with wide-orifice serological pipettes. The RA was added fresh in the medium each time.

### Bisulfite genomic sequencing and COBRA

2 µg genomic DNA were digested with Kpn I (NEB) and treated with sodium bisulfite as described [50]. The treated DNA was then PCR amplified with the following primers: **DP&PP:** oct fw (−208) 5′-TTTGAAGGTTGAAAATGAAGTTTT-3′, oct rev(+55) 5′-CAACCATAAAAAAAATAAACACCCC-3′; **I3:** oct fw(−485) 5′-GTTGTTTTGTTTTGGTTTTGGATAT-3′, oct rev(−235) 5′-AATCCTCTCACCCCTACCTTAAAT-3′; **I2:** oct fw(−848) 5′- AGGTTTTTTTGATTTGAAGTAGA-3′, oct rev(−535) 5′-AACTCTACACCATAAAACCCC-3′; **PE:** oct fw (−1199), 5′-AGGGTAGGTTT TTGTATTTTTTTT-3′, oct rev(−983) 5′-ACTCCCCTAAAAACAACTTCCTACT-3′; **I1:** oct fw (−1670) 5′-GTGTTATGTGTAGTTGTGTGTAGGT-3′, oct rev(−1341) 5′-TTATCTATCTACTCCTACACCATACT-3′; **DE:** oct fw (−2088) 5′-GGTTTTAGAGGTTGGTTTTGGG-3′, oct rev(−1749) 5′-CATCTCTCTAACCCTCTCCATAAATC-3′. The PCR reactions were performed with the FastTaq from Roche. The cycling conditions were: 1 minute at 92°C, followed by 35 cycles of 30 seconds denaturation at 92°C, annealing for 30 seconds and elongation at 72°C for 30 seconds. The exact annealing temperatures for each primer pair were: **DP&PP:** 63°C**, I3:** 58°C, **I2:** 60°C, **PE**: 60°C, **I1:** 60°C and **DE:** 63°C. The PCR products were cloned and approximately 8–30 clones were sequenced for each amplicon. To avoid clonal amplification of the sequences, the transfected cells were not incubated before plating.

Analysis of the sequenced results was performed with the software BiQ Analyser [51]. In brief, the original genomic sequence was aligned with the sequenced clones and the quality of the sequences was assessed. The efficiency of the bisulfite conversion was judged by the absence of non-converted cytosines in a non-CpG context and clones with conversion rates below 90% were removed. Similarly, clones that shared homology with the genomic sequence below 80% or, in rare cases, that had identical (probably clonal) methylation patterns were removed from the analysis. The only exception was the specific case of homogeneously methylated or homogeneously unmethylated clones, which were included in the results.

For the COBRA experiments [52], the bisulfite-treated DNA was amplified as before using the the oct fw (−208) and oct rev(+55) primers. The amplicons were then gel-purified using the QIAquick Gel Extraction Kit (QIAGEN). Purified DNA (10 ng) was digested with HpyCH4 IV (NEB) according to the manufacturer's instruction. The digestion products were resolved in either a 3% agarose gel or a 6% native acrylamide gel and visualized with SYBRGold (Molecular Probes). The gel was scanned in a STORM imaging system (GE Healthcare) at 100 µm resolution. The quantification of the digestion products was performed using the Image J software (http://rsb.info.nih.gov/ij/).

### Statistical methods

All statistical analyses were performed with the R programming language. The methylation levels per segment were calculated by averaging the methylation of all CpGs and all clones for each segment.

To assess the significance of the differences in methylation between segments, the exact Wilcoxon test and the Wilcoxon permutation test was performed using the package ExactRankTests. For these tests the methylation data for each time point were organized in tables were columns represented segments and rows the sequenced clones. The tests were subsequently performed for pairs of columns. Only the comparisons that passed both tests were characterized as significant.

For assessing the pairwise standard deviations of neighboring CpGs in the Lsh KD and controls, first the average methylation of each CpG was calculated from all clones and then the standard deviations were determined. The significance of the different distributions of the standard deviations was assessed with the Wilcoxon test.

The randomized *in silico* data in all cases were obtained by generating random deviates for the distribution of the Wilcoxon Signed Rank statistic obtained from a sample with size n repeated x times, where x is the number of CpGs contained in the segment.

## Supporting Information

Figure S1 Analysis of the methylation profile per segment of the *Oct-4* upstream regulatory region in two independent differentiation experiments using WT ES cells (A and B), tail tip DNA (D) or an *in silico* randomly generated methylation pattern (C).
(0.07 MB PDF)

Figure S2 COBRA validation of the bisulfite sequencing results. (A) Position of the HpyCH4 IV diagnostic site in the bisulfite-converted P amplicon. The upper (purple) sequence represents the result of bisulfite when all the CpGs (blue) in the original DNA are methylated. The preserved HpyCH4 IV site is shown in a red box (also see Figure 2,asterisc). The lower (green) sequence has been derived assuming all the CpGs in the original DNA are unmethylated. Note that in this case the HpyCH4 IV site is lost. (B) Representative image of a COBRA experiment. The 210bp fragment has been derived from a methylated CpG in the original sequence while the undigested 270bp fragment indicates lack of methylation. U:unmethylated, M:methylated, L:DNA molecular weight ladder. (C) Quantification of the results from the COBRA experiments (triplicate). The data are juxtaposed with the % methylation of the specific CpG site as measured by bisulfite sequencing. The error bars show the standard error of the mean.
(0.17 MB PDF)

Figure S3 Shutdown of *Oct-4* relative to *Gapdh* during *in vitro* differentiation of KO ES cells.(A) Wildtype cells, the same as in Figure 1C. (B) *Dnmt1* KO cells, one experiment. (C) *Dnmt3a/b* DKO cells, the average of two independent experiments. (D) *G9a* KO cells, the average of three independent experiments; the error bars are the standard error of the mean in all cases.
(0.15 MB PDF)

Figure S4 Methylation of neighboring CpGs is less coordinated in Lsh knockdown cells than in controls. The panel shows raw methylation data of Lsh KD RA6 cells (red) compared with controls transfected with scrambled siRNA (blue).
(0.16 MB TIF)

Text S1 Supplementary Legend to Figure 3.
(0.03 MB RTF)
