# Supplementary figures and images for: Targeting of De Novo DNA Methylation Throughout the Oct-4 Gene Regulatory Region in Differentiating Embryonic Stem Cells
Source: PLoS One. 2010 Apr 1;5(4):e9937. doi: 10.1371/journal.pone.0009937 (PMC2848578; doi:10.1371/journal.pone.0009937)

A

ES

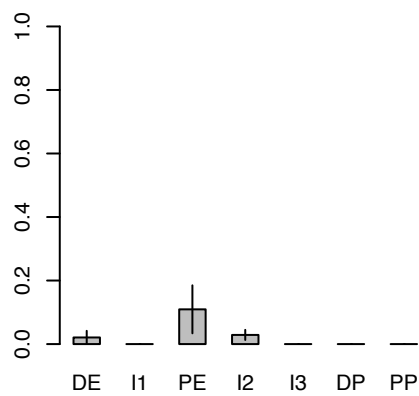

RA4

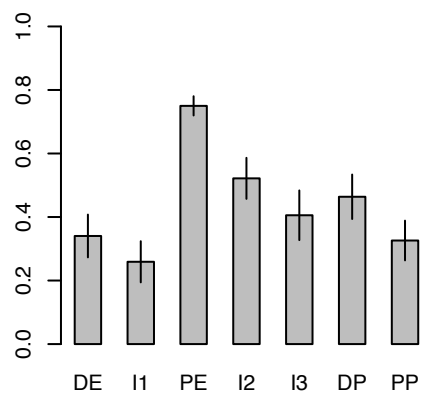

B

ES

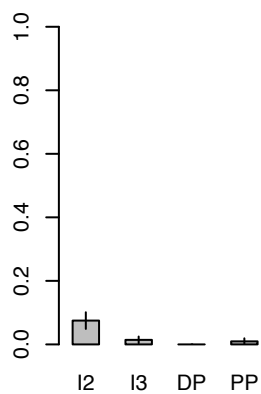

RA4

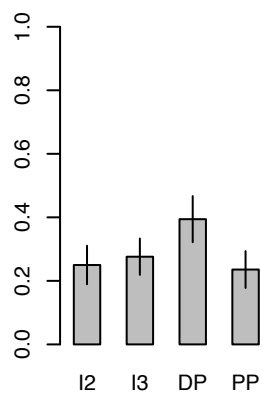

C

RA4

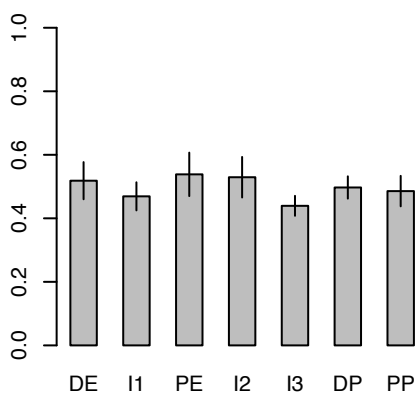

RA6

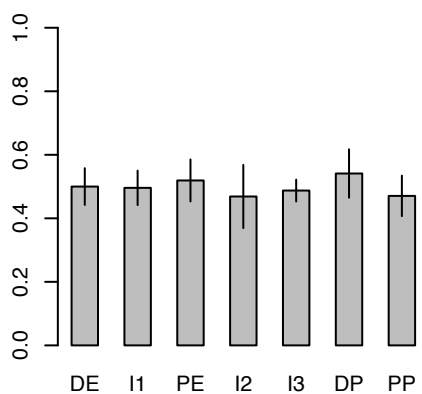

D

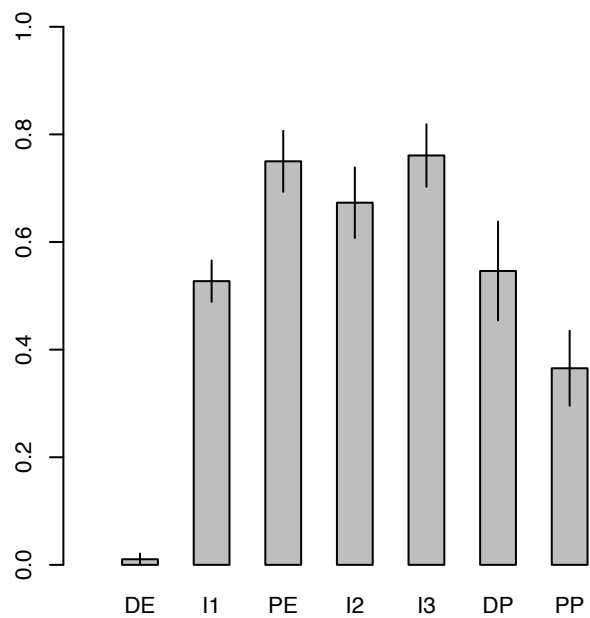

Supplement: Figure S1 — Analysis of the methylation profile per segment of the Oct-4 upstream regulatory region in two independent differentiation experiments using WT ES cells (A and B), tail tip DNA (D) or an in silico randomly generated methylation pattern (C). (0.07 MB PDF) [file pone.0009937.s001.pdf]

**A**

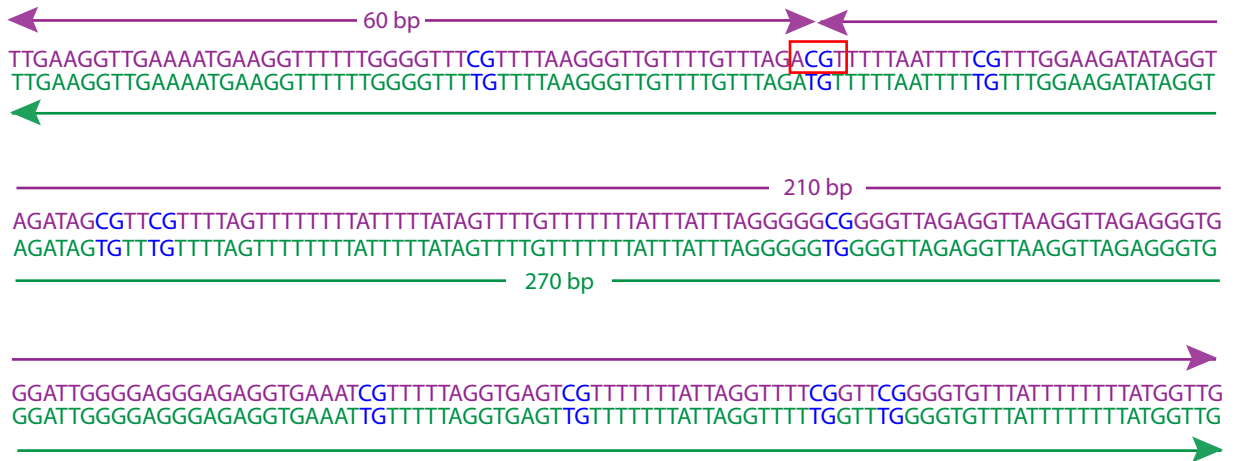

**B**

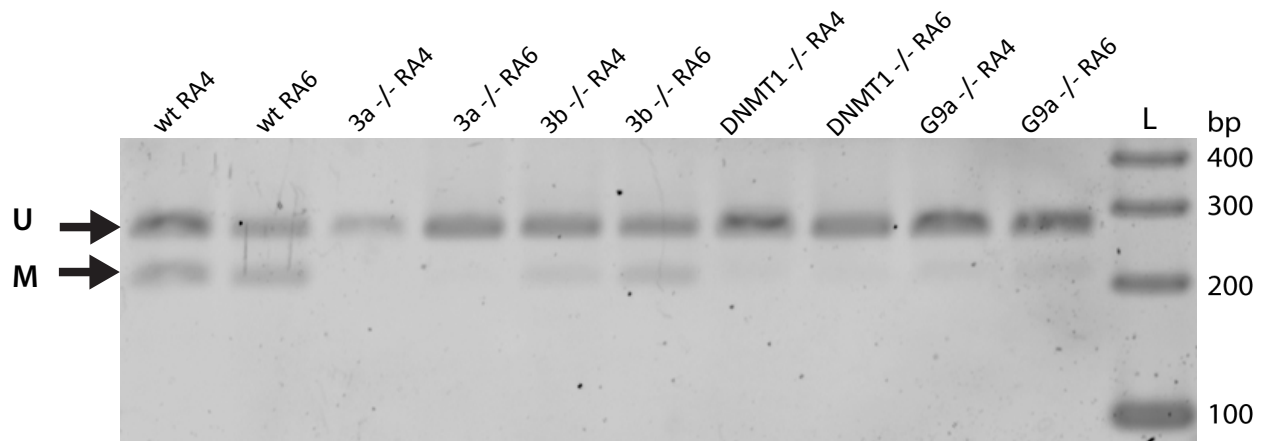

**C**

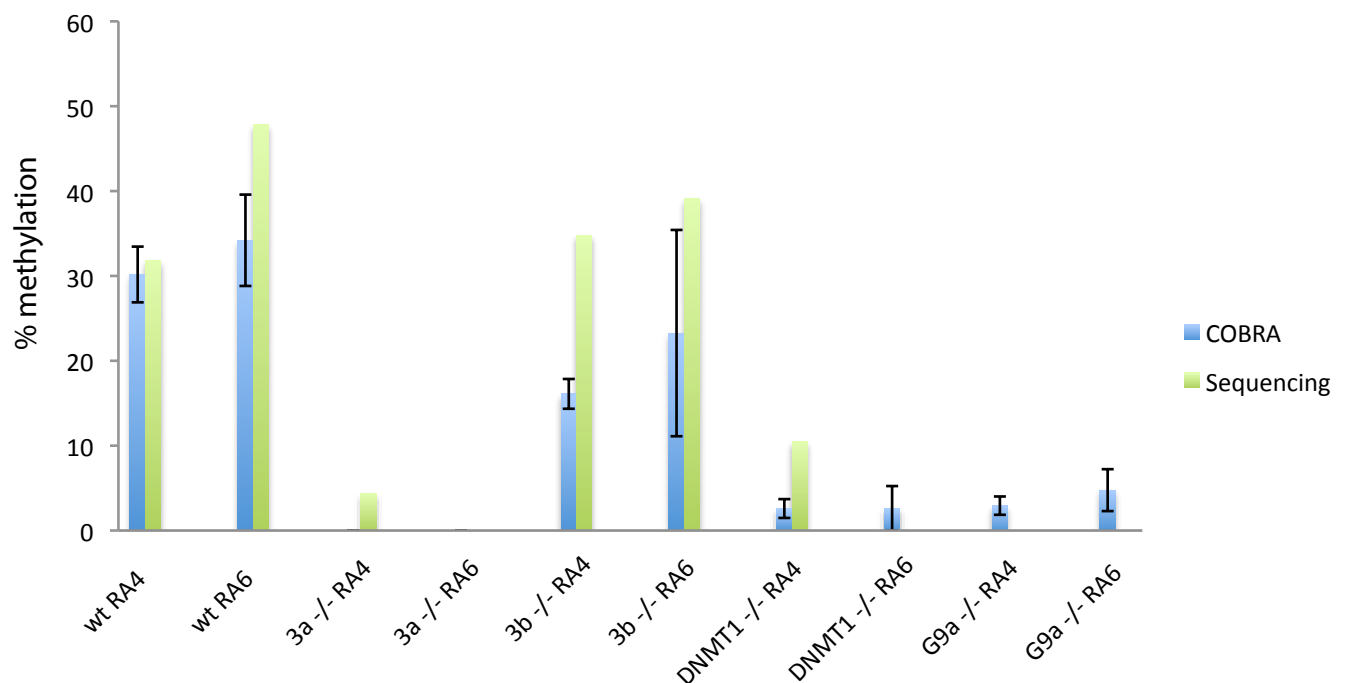

Supplement: Figure S2 — COBRA validation of the bisulfite sequencing results. (A) Position of the HpyCH4 IV diagnostic site in the bisulfite-converted P amplicon. The upper (purple) sequence represents the result of bisulfite when all the CpGs (blue) in the original DNA are methylated. The preserved HpyCH4 IV site is shown in a red box (also see Figure 2,asterisc). The lower (green) sequence has been derived assuming all the CpGs in the original DNA are unmethylated. Note that in this case the HpyCH4 IV site is lost. (B) Representative image of a COBRA experiment. The 210bp fragment has been derived from a methylated CpG in the original sequence while the undigested 270bp fragment indicates lack of methylation. U:unmethylated, M:methylated, L:DNA molecular weight ladder. (C) Quantification of the results from the COBRA experiments (triplicate). The data are juxtaposed with the % methylation of the specific CpG site as measured by bisulfite sequencing. The error bars show the standard error of the mean. (0.17 MB PDF) [file pone.0009937.s002.pdf]

**A**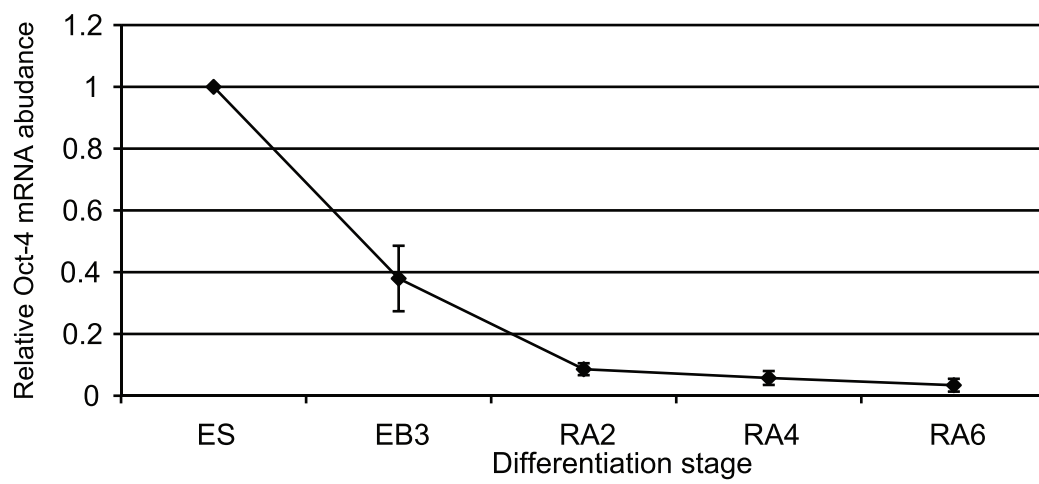**B**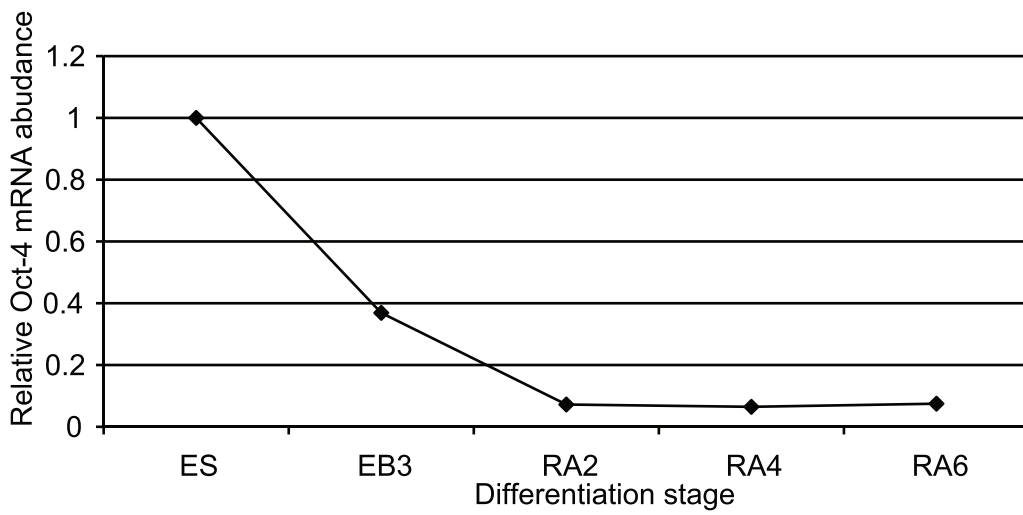**C**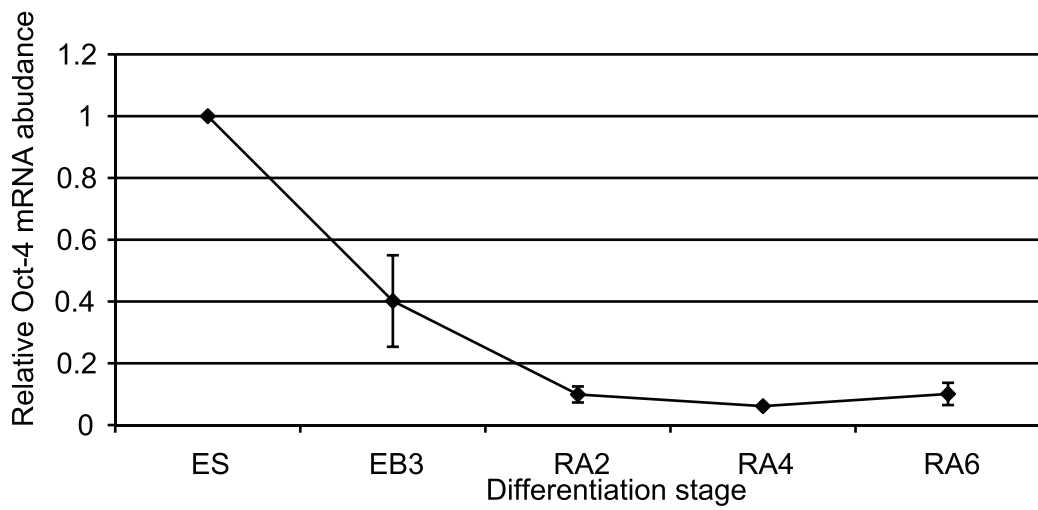**D**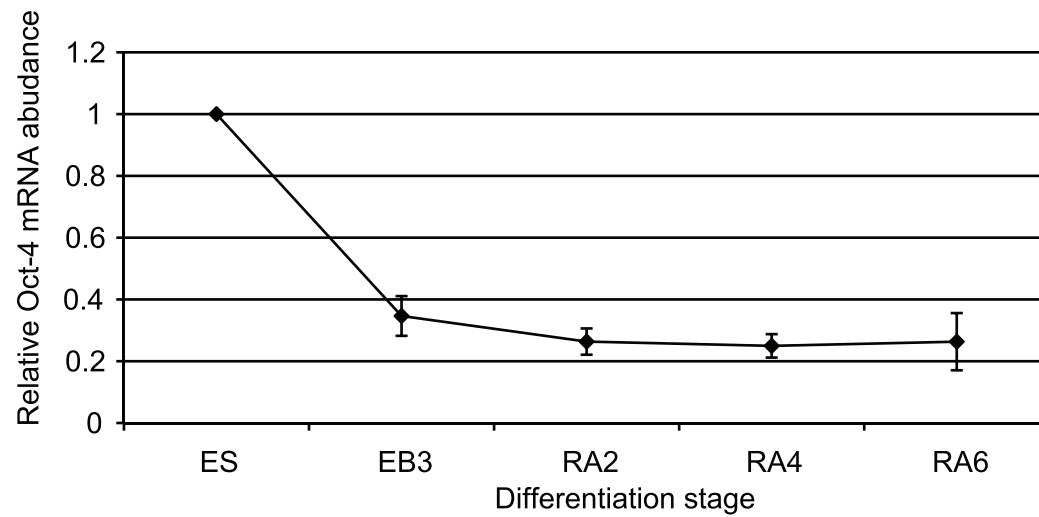

Supplement: Figure S3 — Shutdown of Oct-4 relative to Gapdh during in vitro differentiation of KO ES cells.(A) Wildtype cells, the same as in Figure 1C. (B) Dnmt1 KO cells, one experiment. (C) Dnmt3a/b DKO cells, the average of two independent experiments. (D) G9a KO cells, the average of three independent experiments; the error bars are the standard error of the mean in all cases. (0.15 MB PDF) [file pone.0009937.s003.pdf]

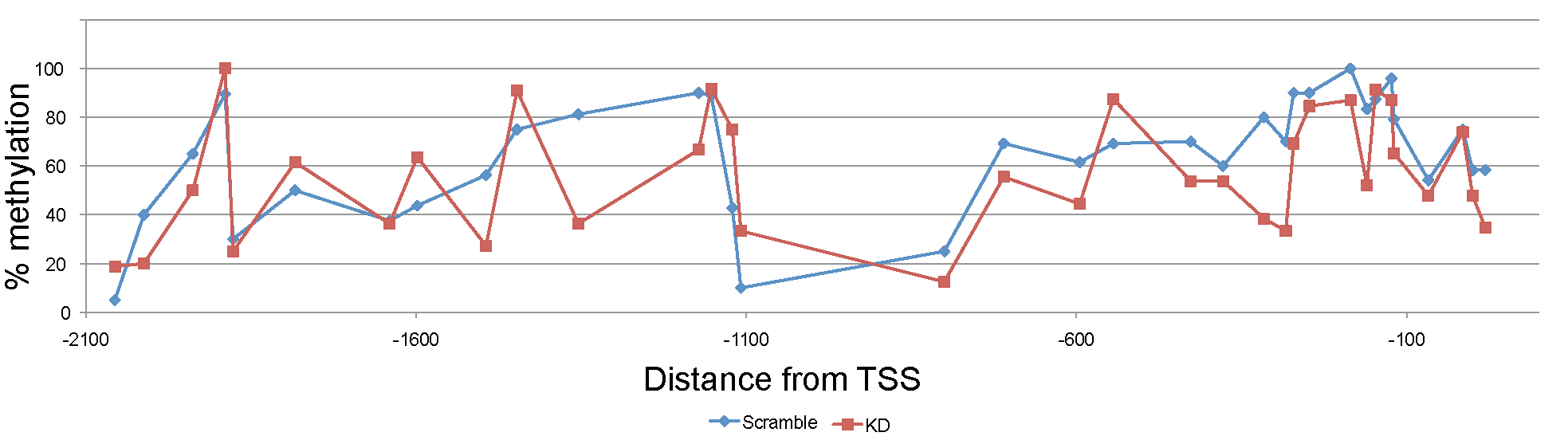

Supplement: Figure S4 — Methylation of neighboring CpGs is less coordinated in Lsh knockdown cells than in controls. The panel shows raw methylation data of Lsh KD RA6 cells (red) compared with controls transfected with scrambled siRNA (blue). (0.16 MB TIF) [file pone.0009937.s004.tif]
