# Supplementary material for: Targeting of De Novo DNA Methylation Throughout the Oct-4 Gene Regulatory Region in Differentiating Embryonic Stem Cells
Source: PLoS One. 2010 Apr 1;5(4):e9937. doi: 10.1371/journal.pone.0009937 (PMC2848578; doi:10.1371/journal.pone.0009937)
Supplement: Text S1 — Supplementary Legend to Figure 3. (0.03 MB RTF) [file pone.0009937.s005.rtf]

Asterisks above individual histogram bars in Figure 3 refer to significant differences in DNA methylation between several other regions. A) In ES the asterisks above I2 compare with all other segments. In RA4 the asterisk above PE compares with DE, I1, I2, I3 and PP. In RA6 the asterisks above DE and PP compare with I1, PE, I2, I3 and DP. B) In RA2 the asterisks above PE compare with all other segments. In RA4 the asterisk above I2 compares with all other segments. In RA4 the asterisks above PE, I2, I3 and PP compare with DE, I1 and DP. C) In RA2 the asterisk above PE compares with I1, DP and PP. In RA4 the asterisks above PE and DP compare with DE, I1, I2 and PP. The asterisk above I3 in the same stage compares with DE, I1 and PP. In RA6 the asterisk above I1 compares with I2 and DP, while the asterisk above PE compares with I1, I2 and PP. D) In RA6, the asterisks above PE and I2 compare with all other segments. E) In RA2 and RA4, the asterisks above PE compare with DE, I1, I3, DP and PP. In RA6 the asterisks above PE compare with DE, I3, DP and PP. F) In RA4, the asterisk above I3 compares with DE, I2, DP and PP.
